# Multi-Object Trajectory Prediction Based on Lane Information and Generative Adversarial Network

**DOI:** 10.3390/s24041280

**Published:** 2024-02-17

**Authors:** Lie Guo, Pingshu Ge, Zhenzhou Shi

**Affiliations:** 1 School of Mechanical Engineering, Dalian University of Technology, Dalian 116024, China; guo_lie@dlut.edu.cn (L.G.); shijia5229350@163.com (Z.S.); 2 Ningbo Institute, Dalian University of Technology, Ningbo 315016, China; 3 College of Mechanical & Electronic Engineering, Dalian Minzu University, Dalian 116600, China

**Keywords:** lane detection, trajectory prediction, channel attention mechanism, generative adversarial network

## Abstract

Nowadays, most trajectory prediction algorithms have difficulty simulating actual traffic behavior, and there is still a problem of large prediction errors. Therefore, this paper proposes a multi-object trajectory prediction algorithm based on lane information and foresight information. A Hybrid Dilated Convolution module based on the Channel Attention mechanism (CA-HDC) is developed to extract features, which improves the lane feature extraction in complicated environments and solves the problem of poor robustness of the traditional PINet. A lane information fusion module and a trajectory adjustment module based on the foresight information are developed. A socially acceptable trajectory with Generative Adversarial Networks (S-GAN) is developed to reduce the error of the trajectory prediction algorithm. The lane detection accuracy in special scenarios such as crowded, shadow, arrow, crossroad, and night are improved on the CULane dataset. The average F1-measure of the proposed lane detection has been increased by 4.1% compared to the original PINet. The trajectory prediction test based on D^2^-City indicates that the average displacement error of the proposed trajectory prediction algorithm is reduced by 4.27%, and the final displacement error is reduced by 7.53%. The proposed algorithm can achieve good results in lane detection and multi-object trajectory prediction tasks.

## 1. Introduction

Realizing the strategic requirements of a strong transportation country and creating an intelligent and safe transportation environment requires continuous improvement and development of key technologies for intelligent vehicles [1,2]. Environment perception can understand the surrounding environment and extract specific locations of the surrounding obstacles, which can provide information for intelligent vehicles to make safety decision control.

Trajectory prediction can predict the future state of nearby obstacles based on their current and previous observations of the surrounding environments [3]. If the intelligent vehicle can predict the future trajectory of the previous vehicles, it can plan a safety path in advance, which can avoid some traffic accidents caused by emergency stops and sudden turns. Therefore, it is necessary to predict the location of the targets around the vehicle and make a prediction of possible collision in advance for intelligent vehicles [4,5].

The rest of the paper is organized as follows. Related works are introduced in Section 2. Section 3 presents a lane detection method based on an improved PINet model. Section 4 proposes a multi-object trajectory prediction based on lane information. Section 5 verifies the performance of the proposed lane detection and trajectory prediction method. The conclusion is given in Section 6.

## 2. Related Works

Deep learning has effectively mitigated several limitations of the traditional lane detection methods based on machine vision, thereby enabling the realization of end-to-end lane detection. For example, He et al. [6] proposed a lane detector using a double-view convolutional neural network. Li et al. [7] used a multi-task deep convolutional neural network to detect the presence of lane lines and their properties relative to the region of interest. Neven et al. [8] proposed an end-to-end LaneNet network to improve the robustness of lane detection using a learnable perspective transformation. Gansbeke et al. [9] proposed a direct regression lane detection algorithm, including a segmented depth network of weight maps and a miniaturizable least squares fitting module. PINet [10] predicted lane lines using key points. It has a shared feature extraction layer and multiple branches for cluster lane detection and embedding, which generates accurate points on the lanes and reduces the clustering problem of the generated points to a point cloud instance segmentation problem. Until now, PINet has shown better lane detection performance compared to other methods under different scenarios. Therefore, this paper chooses the PINet model as the fundamental model to realize lane detection. Traditional object prediction methods use only their current states or history trajectories of their states over time. Most methods begin with the trajectory prediction for pedestrians. For example, Morris et al. [11] applied Gaussian mixture models to achieve trajectory clustering, facilitating the learning of points of interest, and employed Hidden Markov models to probabilistically encode diverse pedestrian behaviors. Subsequent research endeavors utilized a range of hand-crafted features to enhance trajectory prediction accuracy. However, these models are hindered by limited generalization capabilities, particularly in adapting to traffic scenarios featuring intricate social dynamics. Deep learning methodologies, renowned for their effectiveness in navigating complex environments, have emerged as preferred alternatives due to their superior performance. Among them, Recurrent Neural Network (RNN) and its variants, Long Short-Term Memory (LSTM) and Gated Recurrent Units (GRU), have been very successful in sequence prediction tasks [12].

For pedestrian trajectory prediction, Social-LSTM [13] was proposed by Stanford University for human trajectory prediction. However, Social-LSTM has to run the LSTM once for each target, which requires a high computational cost and cannot perform a global consideration of all targets. Socially acceptable trajectories with Generative Adversarial Networks (Social GAN) [14] introduced a social pooling module between encoder and decoder. It has the ability to learn the surrounding interaction information between pedestrians. Therefore, it only needs to go through the LSTM once, reducing the computational cost. However, most existing methods cannot fully extract the interaction information between pedestrians. Fang et al. [15] proposed an attention mechanism based on GAN to model the social relationship of interaction information between pedestrians. Sighencea et al. [16] studied a deep learning-based pedestrian trajectory prediction method. Wang et al. [17] proposed a multi-information-based convolutional neural network combining pedestrian pose and 3D spatial information to identify pedestrian’s intent. A depth map containing 3D geometric information around the pedestrian was constructed to predict its trajectories. Lu et al. [18] proposed a heterogeneous context-aware graph convolutional network based on an encoder-decoder architecture. They exploited the spatiotemporal evolution of interaction patterns and captured high-fidelity interaction contexts. However, the model does not introduce a rich infrastructure and does not extend to pedestrian-vehicle interaction.

For vehicle trajectory prediction, Zyner et al. [19] used a three-layer LSTM of codes to predict the parameters of a weighted Gaussian mixture model for each future step of the vehicle. Ding and Shen [20] used an encoder LSTM to predict the intention of the target vehicle using its state. Dai et al. [21] used two LSTM networks for target vehicle trajectory prediction. One network was designed to model the trajectory of the target vehicle and surrounding vehicles individually. The other network was designed to model the interaction between the target vehicle and each surrounding vehicle. Diehl et al. [22] enhanced vehicle trajectory prediction by comparing and improving graph convolution networks and graph attention networks. Lin et al. [23] analyzed the spatiotemporal attention weights of vehicles based on vehicle class, location, and traffic density, increasing the interpretability of the trajectory prediction model. Messaoud et al. [24] introduced a trajectory prediction method based on vehicle interaction modeling, using an attention mechanism to emphasize the influence of surrounding vehicles on the future of the main vehicle. A dual-attention mechanism trajectory prediction model based on LSTM was proposed by Guo et al. [25]. To achieve higher trajectory prediction accuracy, the spatial attention mechanism combines the self-vehicle motion trend with the free space of surrounding vehicles, while the temporal attention mechanism maximizes the use of historical trajectory information. Because existing studies focus on the spatial interactions of adjacent vehicles regardless of the time dependence. Jiang et al. [26] proposed a spatiotemporal attention LSTM encoder-decoder model to predict vehicle trajectories. Salzmann et al. [27] proposed a modular, graphically structured recurrent model capable of combining dynamic and heterogeneous data from agents and making different predictions depending on the scene structure. The method can generate predictions for different trajectories conditional on the target vehicle and agent motions and can demonstrate its performance on real trajectory prediction datasets, outperforming a range of state-of-the-art deterministic and generative methods. Other methods extract features directly from traffic map scenes to make predictions of vehicle trajectories, such as [28], which proposed an ambient attention network that used a graph attention network to extract scene features, thus maintaining the spatial relationship between vehicles and scene structure. Zhong et al. [29] constructed a generative model framework using an auto-encoder to dynamically predict the future trajectories of surrounding vehicles.

The current trajectory prediction is mainly for a single target of pedestrians or vehicles. However, the motions of vehicles, pedestrians, bicycles, motorcycles, etc., are often interrelated with each other under real traffic scenarios. Considering the interactions of only one class object may generate prediction errors. Current trajectory prediction algorithms seldom consider the semantic information from the traffic environment, which leads to the situation that the prediction accuracy is more objective in a short period of time by only relying on the historical trajectory information. Meanwhile, the prediction accuracy is insufficient for a long period of time. Employing analysis of the cues that traffic participants travel in the actual traffic scenes, lane information has a relatively large influence on those traffic participants, and most of their movements will follow the lanes. The traffic participants themselves can adjust their trajectories by predicting the positions of other targets in the surrounding environment. For this reason, this paper improves the accuracy of long-time prediction by adding lane information to realize multi-object prediction.

In summary, this paper has the following main contributions: (1) A lane detection based on an improved PINet model is proposed by combining Hybrid Dilated Convolution and Channel Attention mechanism (CA-HDC). The proposed lane detection method can improve the accuracy of lane detection under different traffic scenarios. (2) A multi-object trajectory prediction method has been presented by adding a lane information fusion module and a trajectory adjustment module. The proposed trajectory prediction method enables the network to consider the high-level semantic information around the target more fully and enhance the prediction performance. (3) Comparative experiments with mainstream detection and prediction methods have been conducted to fully show the advancement of our method under different datasets.

## 3. Lane Detection Based on Improved PINet Model

To achieve accurate lane detection under real traffic scenarios, this paper improves the traditional PINet model to realize lane detection. A combination of HDC and ordinary convolution layers is utilized to extract features. A channel attention mechanism is utilized to select the extracted features by itself, and the extraction of features with different perceptual fields is conducted to improve the accuracy of lane detection under different traffic scenarios.

### 3.1. Feature Extraction

#### 3.1.1. Hybrid Dilated Convolution Module Based on Channel Attention

The Max pooling mechanism and the Gate mechanism can be approximated as saliency-based and bottom-up attention mechanisms. For example, SEnet [30] boosts and suppresses features that are not important for the current task by obtaining the importance of each feature channel in the network. The Convolutional Block Attention Module (CBAM) [31] adds max pooling to SEnet. Spatial attention is achieved by performing maximum and average pooling on a channel-by-channel basis and concatenating the results of both, followed by a convolutional reduction to a 1 × w × h feature map spatial weight, which is then dotted with the input features to implement the spatial attention mechanism. This paper adopts the channel attention extraction method of CBAM to extract the channel attention.

The structure of HDC can solve the pixel point loss problem that occurs by using cavity convolution continuously. The HDC should ideally satisfy the following equation.

(1) $$M_{i}=\max\left[M_{i+1}-2r_{i},M_{i+1}-2\left(M_{i+1}-r_{i}\right),r_{i}\right]$$

(2) $$M_{2}\leq k$$

where $r_{i\;}$ is the dilation rate at layer i, and $M_{i\;}$ is the maximum void rate at layer $i.{\;M}_{n}$ at the last layer is equal to $r_{n}$ by default, the convolutional kernel of size is k×k.

**Figure 1:**
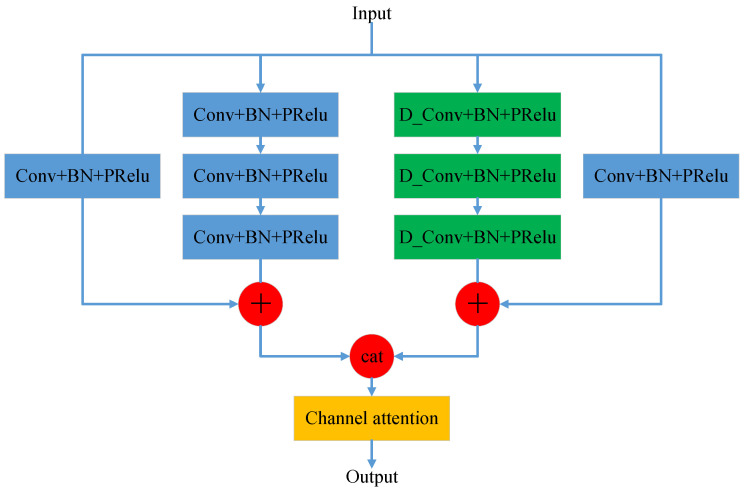
CA-HDC module.

Convolutional neural networks extract features by fusing spatial information and channel information via convolutional operations. This paper enhances the representational capability of the network model from the perspective of enhanced channel dimensional encoding. Channel attention is utilized to model the relationship between channels and to explore the dependency of feature maps on each channel. This can enhance the influence of useful information and suppress useless information. The CA-HDC module is shown in Figure 1.

The CA-HDC module contains three parts. The first normal convolution of the residual block directly connected part contains three convolution kernels [32]. The jump-connected part also goes through a convolution kernel. Each convolution operation is followed by a batch normalization and a PReLU activation function. The residual block of the dilated convolution is similar to the residual block of the normal convolution, except that the normal convolution in the directly connected part is replaced by an HDC module with a different dilation rate. The results of the two parts are obtained and then connected in the channel dimension, and the results are output by a channel attention module for the important selection of feature channels with different field of view ranges.

#### 3.1.2. Resizing Layer of the CA-HDC Module

The resizing layer has two functions. Firstly, it can realize the initial extraction of features from the input image. Secondly, it helps to reduce the size of the feature map so that the later instance segmentation by the hourglass network does not have to revert to the original image size. Therefore, it can reduce the computational effort of instance segmentation. The resizing layer consists of one general convolution, three CA-HDC modules, and two maximum pooling layers.

The whole network consists of a resizing layer to initially extract features and reduce the feature map to reduce the amount of computation for lane segmentation. The hourglass network then further extracts the semantic information of the image, and the final three detection branches output the final detection results. A 512 × 256 image is input to the network, and the 64 × 32 feature map is output after the resizing layer. Since the hourglass network and the prediction branch do not change the size of the feature map, the final output feature map size is also 64 × 32. The confidence branch outputs the predicted confidence value of each grid, and the feature map size is set to be 64 × 32 × 1. Since the predicted x-axis offset and y-axis offset are to be output, the feature map size is set to be 64 × 32 × 1. The number of feature maps in the feature branch does not have much influence and is set to be 4 in this paper.

#### 3.1.3. Hourglass Network

**Figure 2:**
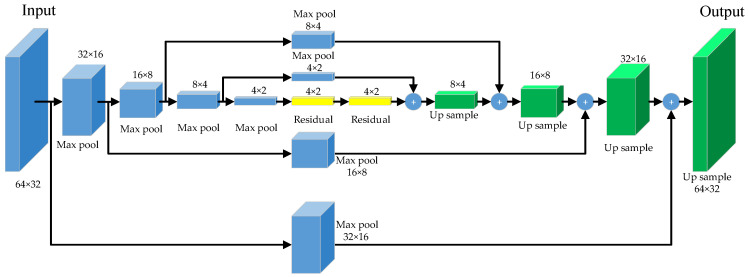
Hourglass network.

After the image is resized, the size of the feature map is 64 × 32, and it enters the hourglass network for feature extraction. The hourglass network can present a symmetric distribution of capacity between bottom-up processing (from high resolutions to low resolutions) and top-down processing (from low resolutions to high resolutions) [33]. In each Hourglass module, residual module and max pooling are used to reduce features to a very low resolution. As shown in Figure 2, the blue part indicates the down-sampled feature map by using max pooling, the green part indicates the up-sampled feature map by using the nearest neighbor interpolation, and the yellow part indicates the feature map with the same size. The left and right parts are mirrors of each other, and the resolution of the down-sampling image is reduced to half each time it passes through each bottleneck structure. The bottleneck structure has three convolutional layers used for the direct connection and one convolutional layer used for the jump connection. The resolution of the up-sampling image is doubled each time when it passes through each bottleneck structure. The feature maps at the corresponding positions are of the same size. The feature maps can contain more semantic and detailed information after being added together.

### 3.2. Lane Detection

**Figure 3:**
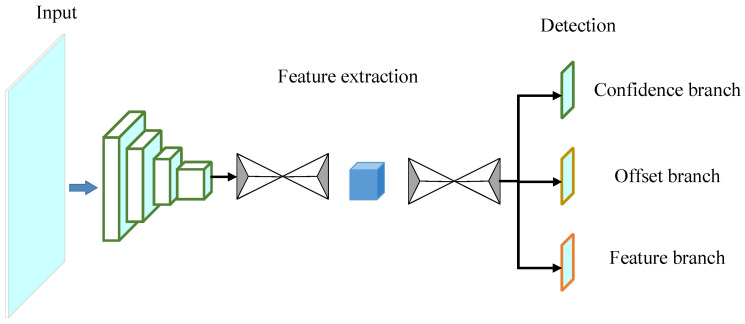
Structure of lane detection network.

After the feature extraction layer completes the feature extraction, the detection branch is used to perform the location prediction of key points and the instance segmentation of lines. The overall structure of the lane detection network is shown in Figure 3.

The detection branch consists of three components: the confidence branch, the offset branch, and the feature branch. The confidence branch outputs the confidence value for each grid, which is used to predict whether there is a key point of lane in that grid. Due to the reduction in feature maps, for instance, segmentation to ensure the calculation speed, an offset output value is needed to enable the key points to be accurately reflected in the original map. The two channels of the offset branch output are used to predict the x-axis offset and y-axis offset, respectively. The feature branch output is used to separate key points belonging to different lane lines and is trained to bring the values of key points representing the same instance closer together.

The input image is initially extracted features by the resizing layer. The feature map is reduced to save the computational complexity of lane instance segmentation. The hourglass network further extracts the semantic information of the image, and the three detection branches output the final detection results. An image with a size of 512 × 256 is input to the lane detection network. A size of 64 × 32 feature map is output after the resizing layer. Since the hourglass network and the prediction branch do not change the size of the feature map, the final output feature map size is also 64 × 32. The detailed structure of the lane detection network is shown in Table 1.

## 4. Multi-Object Trajectory Prediction Based on Lane Information

### 4.1. S-GAN Trajectory Prediction Model

S-GAN combines a sequence-to-sequence prediction model and a generative adversarial network to handle multimodal pedestrian trajectory prediction. The function of predicting the future trajectory of pedestrians is achieved by observing its historical trajectory information. A multilayer perceptron and a pooling mechanism are utilized to extract information about social relationships between each pedestrian. S-GAN adds a generative adversarial network to the sequence prediction. It can make the prediction results diverse and multimodal using iterative adversarial training of generators and adversaries. On this basis, this paper incorporates the lane information and foresight mechanism to improve the accuracy of the multi-object trajectory prediction algorithm.

**Figure 4:**
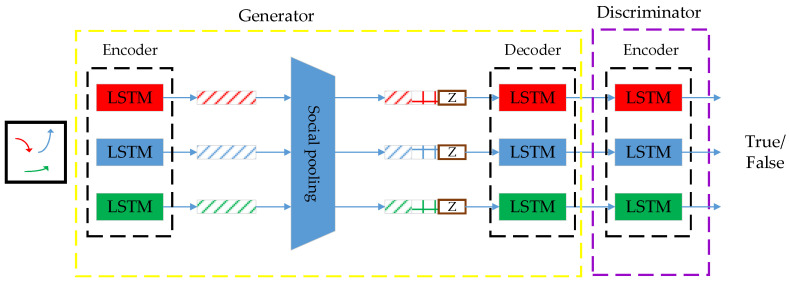
Framework of the S-GAN model.

S-GAN generally consists of two adversarial sub-models, the generator, and the discriminator, as shown in Figure 4. The generator has an encoder-decoder structure responsible for generating a sample based on a random noise input. The discriminator is a multilayer perceptron that determines whether the input sample is a real or false sample generated by the generator.

The generator inputs the historical relative trajectories of all traffic participants within a continuous sequence, with a certain time step into the LSTM for encoding. It extracts the historical features of each target. Since the current position of each target depends not only on its present position and historical state but also on its social relationships with other targets, the relationship between each target is primarily based on its relative distance. The relative distance between each target in the current frame is calculated to obtain social relationship information using a multilayer perceptron. After the social pooling layer aggregates the historical trajectory information, social relationship information, and random noise, each sequence is input to the LSTM decoder for decoding. The predicted trajectories are output sequentially according to a certain time step. The discriminator determines whether the input trajectory is the real trajectory or the false sample generated by the generator. Via the mutual game between the generator and the discriminator, the parameters of the generator are continuously adjusted to make the generated trajectory conform to the real trajectory as much as possible.

### 4.2. Lane Information Fusion Module

**Figure 5:**
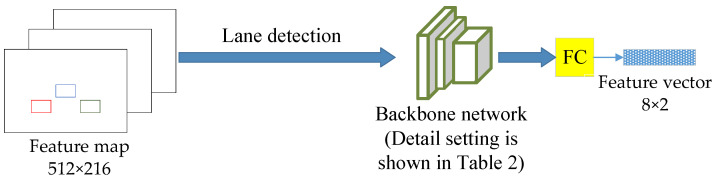
Lane information fusion module.

Under real traffic scenarios, each participant is influenced by other participants and the environment. The future location of the vehicle and pedestrian can be predicted based on the information from the traffic scene, especially the lane information. The trajectory of the target will not change much in a short prediction period. Therefore, the error in predicting future trajectories based on historical trajectories over a short prediction period is not likely to be significant. However, over a longer prediction period, the trajectory of the target may be influenced by changes in the environment. Nevertheless, such changes are typically constrained by lane information. For this reason, a lane information fusion module is designed to enhance the prediction capability of the network by considering the high-level semantic information around the target, as shown in Figure 5.

Table 2 shows the details of the lane information extraction backbone network. The lane detection algorithm is used to extract the lane information into a 512 × 256 feature map, which is dimensionally reduced by a backbone network consisting of three general convolutional layers: a BatchNorm layer, a Relu layer, and a maximum pooling layer. For faster information extraction, all the general convolutional layers use eight convolutional kernels to abstract the feature maps into a size of 32 × 16 feature maps layer by layer. The maximum pooling layer is used to reduce the dimensionality of the feature map extracted from the convolutional layer, and a size of 16 × 8 feature map can be obtained. Finally, the feature vectors with the size of 8 × 2 are extracted by a fully connected layer as the input to the decoder of the trajectory prediction model.

### 4.3. Trajectory Adjustment Module Based on Foreseeable Information

Usually, the driver will take into account the movement of the surrounding objects and consider the possible collision with other traffic participants. Therefore, a trajectory adjustment module is designed based on some foreseeable information according to the previously predicted trajectory by reasonable use of future information to adjust the current predicted trajectory.

**Figure 6:**
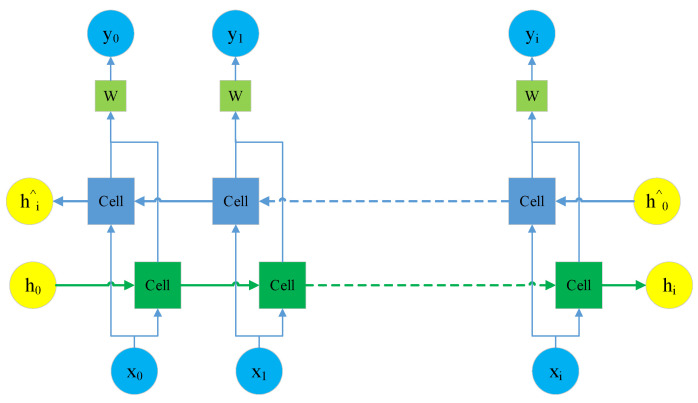
Trajectory adjustment module.

A bidirectional LSTM is utilized to perform the prediction. The forward LSTM conducts the prediction based on the vehicle dynamics information. In contrast, the backward LSTM adjusts the results by its foresight ability. The input data are the predicted trajectory information. The social relationship information of the vehicle is added at each time step. The neurons of the bidirectional LSTM output two results for each time step, and a learnable coefficient is used to give the importance evaluation in both directions, as shown in Figure 6. In Figure 6, *x_i_* and *y_i_* represents the input and output variables of each step *i*, respectively. *W* represents the proportion of forward predicted in the final prediction. *h_i_* and *h^^^_i_* represents the forward and backward prediction of each step *i*, respectively.

The final prediction results are:

(3) $$Y=W\times Y_{f}+\left(1-W\right)\times Y_{b},$$

where *Y* represents the output, *W* represents the proportion of forward predicted in the final prediction, *Y*_f_ represents the forward prediction result, and Y_b_ represents the backward prediction result. The two prediction directions work together to improve the prediction accuracy.

### 4.4. Multi-Object Trajectory Prediction Model Based on S-GAN

**Figure 7:**
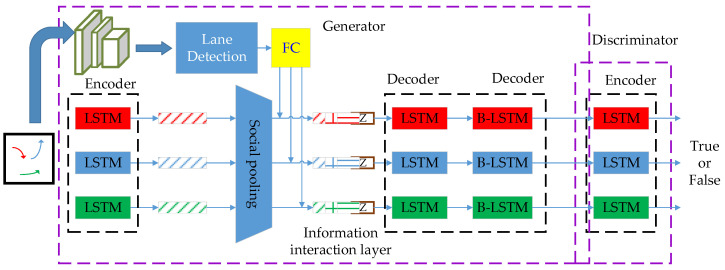
Framework of multi-object trajectory prediction model based on S-GAN.

The proposed multi-object trajectory prediction model based on S-GAN is shown in Figure 7, which consists of the generator and the discriminator. The task of the generator is to predict the future trajectory Y_i_ based on the historical trajectory X_i_ in the past period, while the discriminator learns to identify whether the input trajectory is a trajectory from the real dataset [X_i_,${\hat{Y}}_{i}$] or a false trajectory generated by the previous generator [X_i_,${\tilde{Y}}_{i}$].

Figure 7 shows the trajectory prediction model when there are three targets in the scene. The generator includes three parts: an encoder, an information interaction layer, and a decoder. The encoder receives the historical position X_i_ of the current target and distills the historical trajectory information $h_{i}^{t-1}$ through LSTM to participate in the next operation. The information fusion layer receives the relative position $R_{i}$ of other individuals relative to the current individual and the historical trajectory information $h_{i}^{t-1}$ obtained through the encoder, fuses the lane information $H_{i\;}^{l}$ proposed through the convolutional network and adds a Gaussian noise z. The decoder accepts the state $h_{t}^{i}$ of the current target from the information interaction layer, initializes the LSTM, and takes the position of the previous moment as the input to decode the corresponding predicted trajectory information. Then, the final predicted trajectory $\tilde{Y}$ is obtained by the trajectory adjustment module based on the foreseen information. The structure of the discriminator is similar to that of the encoder in the generator.

#### 4.4.1. Encoder

The input of the encoder module LSTM is the position of the target at the current moment $X_{i}^{t}$. LSTM focuses more on the velocity variation, and each target has its own motion characteristics. Motion characteristics are the most basic prediction information in the trajectory prediction task and reflect the observable physical properties of the target. The original S-GAN model does not include the dimensional information of the predicted targets, while this paper adds two attributes: the center position (x_c_, y_c_) and the width and height size (w, h) of the target vehicle. To obtain the motion information of each vehicle, this paper converts the trajectory sequence from coordinate space to feature space by a fully connected layer to obtain the motion features $E_{i}^{T-T_{obs}:T}$ from *T* − *T_obs_* time to time *T*.

(4) $$E_{i}^{T-T_{obs}:T}=\varphi \left(X_{i}^{T-T_{obs}:T},W_{e}\right),$$

where φ is the embedding vector used for feature transformation and $W_{e}$ is the weight of this vector, $X_{i}^{T-T_{obs}:T}$ denotes the individual target motion information from time *T* − *T_obs_* to time *T*.

The obtained embedding vectors of each time step will be input to the LSTM, and the encoded motion feature vector $h_{i}^{t}$ can be obtained.

(5) $$h_{i}^{t}=LSTM\left(E_{i}^{T-Tobs:T},h_{i}^{t},W_{l}\right),$$

where $h_{i}^{t}$ is the hidden state of the LSTM for vehicle *i* at time step *t*. This hidden state reflects the motion characteristics of vehicle *i* during the first *t* time step.

The motion information encoded by the neural network contains not only the position and size information of the target but also the change of position that reflects the direction of the target’s motion and the change of size that reflects the pose of the target and the distance from the host vehicle.

#### 4.4.2. Information Interaction Layer

In congested traffic scenarios, the movement of a target is influenced by the surrounding participants, which leads to steering, following, accelerating, or decelerating of the host vehicle. This influence is often mutual and is called social information, which is an important factor influencing the future trajectory of vehicles. The information interaction layer uses maximum pooling to obtain social information among targets. All target hidden states *h^t^* in the same frame with the target frame (x, y, w, h) of the target in that frame are fed into this social pooling module. The distance between each target is calculated using the target position size of that frame.

(6) $$\left(x_{r},y_{r},w_{r},h_{r}\right)=(x_{1}-x_{2},y_{1}-y_{2},w_{1}+w_{2},h_{1}+h_{2}),$$

where *x* represents the horizontal coordinate of the center point of the target, *y* represents the vertical coordinate of the center point of the target, *w* represents the width of the target frame, and *h* represents the height of the center point of the target. (x_r_, y_r_, w_r_, h_r_) represents the target position size of the predicted target, (x_2_, y_2_, w_2_, h_2_) represents the target position size of the target around the predicted target, and (x_1_, y_1_, w_1_, h_1_) represents the relative position size between the predicted target and the relative target.

The lane position in each frame can be achieved according to the lane detection model. The key points of the lane are embedded in the feature map. Then, the lane information is extracted using a convolutional neural network consisting of three consecutive convolutional layers: a BatchNorm layer, a Relu layer, and a Max Pooling layer. The feature extraction network first extracts lane information, which is then fed into the information interaction layer via a fully connected layer. Subsequently, a multilayer perceptron is employed to fuse historical trajectory information, target interaction information, and lane-target interaction information, creating the input for the decoder.

#### 4.4.3. Decoder

The decoder receives the fused information from the information interaction layer, along with the last target information in the previous frame. The coordinate information is embedded into a feature vector by a fully connected layer with the relative position and size change of the target.

(7) $$E_{i}^{t}=\varphi \left(X_{i}^{t},W_{e}\right)$$

where $X_{i}^{t}$ denotes the last position and size of the detected target, φ is the embedding vector used for feature transformation and $W_{e}$ is the weight of this vector.

**Figure 8:**
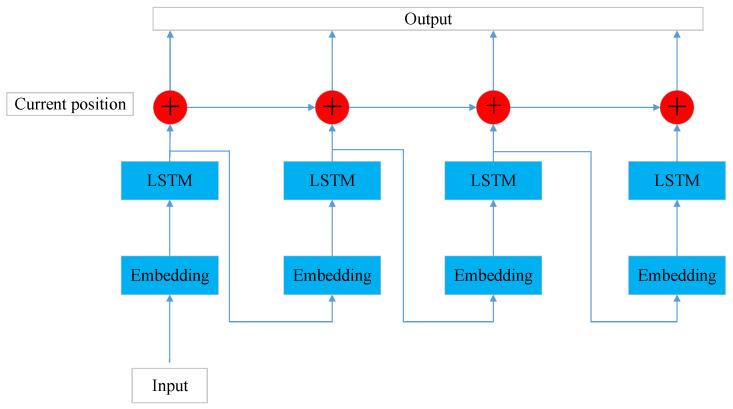
Decoder.

Then, the results are generated step by step in the decoder, as shown in Figure 8. LSTM receives the relative feature vector of each step as input. The fusion information generated in the information interaction layer is used as a hidden unit to generate the relative position and target size change of the current prediction step. Then, the generated results are conducted into the LSTM of the next step. The feature embedding through the fully connected layer is required before conducting into the LSTM. This process continues until the relative position and frame size change for all predicted steps are generated. Finally, the position and size of the target from the last frame of the historical trajectory are added to the relative position and size of the predicted target for each prediction step to obtain the final result.

The loss function contains generator loss, discriminator loss, and L2 loss of the prediction results. L2 loss of the prediction results and the generator loss function are used for the training of the generator, and the discriminator loss function is used for the training of the discriminator.

(8) $$L_{G}=\bar{Y}-Y\times Y^{\wedge }+\log\left(1-Y\right),$$

where *L_G_* denotes the generator loss, Y denotes the discriminator score of the predicted trajectory generated by the generator, $Y^{\wedge }$ denotes the best score, and $\bar{Y}$ denotes the Y that its lower bound is not exceeding 0. This loss function is a cross-entropy loss function that can be numerically stable.

(9) $$L_{D}=\left(\bar{Y}-Y\times Y^{\vee }+\log\left(1-Y\right)\right)+\left(\bar{\tilde{Y}}-\tilde{Y}\times {\tilde{Y}}^{\wedge }+\log\left(1-\tilde{Y}\right)\right),$$

where *L_D_* denotes the discriminator loss, $\tilde{Y}$ denotes the score of the true trajectory on the discriminator, $Y^{\vee }$ denotes the worst score, ${\tilde{Y}}^{\wedge }$ denotes the best score, and $\bar{\tilde{Y}}$ denotes the $\tilde{Y}$ that its lower bound is not exceeding 0. This loss function contains the cross-entropy loss function of the discriminator for both predicted trajectories and true trajectories.

(10) $$L_{2}={\left(X^{\wedge }-X\right)}^{2},$$

where *L*_2_ denotes the L2 loss of the predicted trajectory, $X^{\wedge }$ denotes the true value of the trajectory, and X denotes the predicted value of the trajectory.

## 5. Test Results

### 5.1. Lane Detection Results

CULane is a large-scale, challenging dataset for academic research on traffic lane detection [34]. The CULane dataset is collected from cameras mounted on six different vehicles, with over 55 h of video and 133,235 frames of image. The CULane dataset is divided into 88,880 training sets, 9675 validation sets, and 34,680 test sets. Depending on the scenario, the dataset is also divided into nine traffic scenarios, such as normal, night, crowed, shadow, and dazzle light, which can lead to better robustness of the trained detection models. This paper chooses the CULane dataset to evaluate the lane detection tests. The characteristics of the CULane dataset are shown in Table 3.

For the main evaluation metric of lane detection, the commonly used evaluation metric for the CULane dataset is F_1_-measure, which is a comprehensive evaluation metric given based on both precision (*p*) and recall (*r*). It is defined as:

(11) $$F_{1}=\frac{2rp}{r+p}$$

The evaluation results of the proposed lane detection algorithm on the CULane dataset are shown in Table 4. As can be seen in Table 4, the proposed lane detection algorithm shows better performance on the CULane dataset compared to the original PINet, especially in situations where the field of view is more heavily obscured, such as crowed, shadow, arrow, crossroad, and night scenarios, and the large perceptual field can improve the performance of lane detection. The average F_1_-measure of the proposed lane detection algorithm has been increased from 66.4% to 69.1%, marking a 4.1% improvement compared to the original PINet. Especially, the F_1_-measure for the shadow scenario has been increased from 61.0% to 70.4%, representing a remarkable 15.4% increase compared to the original PINet.

**Figure 9:**
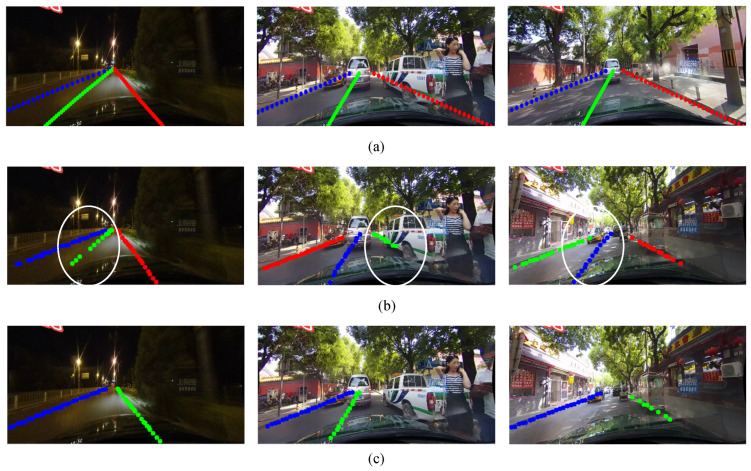
Comparison of lane detection results. (**a**) Real labels. (**b**) Results of the proposed lane detection algorithm. (**c**) Results of the original PINet.

A comparison of the detection result of the proposed lane detection algorithm and the original PINet is shown in Figure 9.

Figure 9 indicates that the proposed lane detection model has improved the detection performance to a certain extent compared to the original PINet model. As can be seen from the white circle in Figure 9b, the proposed lane detection model can detect certain lanes that were undetectable by the original PINet model.

### 5.2. Trajectory Prediction Results

In this paper, the D^2^-City dataset of Didi [35] is selected as the dataset for trajectory prediction tests. D^2^-City dataset is a large-scale comprehensive collection of dashcam videos collected by vehicles on Didi’s platform, which can deeply reflect the diversity and complexity of real-world traffic scenarios in China. The D^2^-City dataset was captured from 5 different cities, with more than 10,000 videos. The images are also labeled with 12 types of targets, including cars, buses, tankers, trucks, bicycles, motorcycles, and pedestrians. Compared with existing publicly available autonomous driving datasets, the image data of the D^2^-City dataset covers more data in complex traffic scenarios, such as road congestion, poor lighting conditions, bad weather, low image clarity, and other scenarios, and includes a variety of traffic environments and weather conditions, which can ensure that our trajectory prediction model has better robustness and can adapt to various conditions. The data are extracted according to the frame rate of 5 FPS, the historical trajectory information of the first 1 s with a total of 5 frames is taken for prediction, and the historical trajectory of the following 2 s with a total of 10 frames is taken for detection.

Average Displacement Error (*ADE*) and Final Displacement Error (*FDE*) are generally used to evaluate the performance of trajectory prediction algorithms [36]. Since the size of the image samples taken at different moments in the D^2^-City dataset is not the same, the normalized *ADE* and *FDE* of the position coordinates are used in order to make the image with different sizes have the same weight in the dataset when they are evaluated.

*ADE* represents the mean square difference between the coordinates of the target’s center point, its width and height, and the position coordinates of the real trajectory, along with the width and height of the target at each step of the predicted trajectory. It can be calculated as follows.

(12) $$ADE=\frac{1}{T_{\mathrm{pred}}N}\sum_{i=1}^{N}\left\|{{\overset{\wedge }{X}}_{i}}^{T:T+T_{pred}}-{X_{i}}^{T:T+T_{pred}}\right\|\times 100$$

where *ADE* is the average displacement error, *T_pred_* is the predicted time step, *N* is the number of targets in the traffic scene, and *T* represents the current time, ${\overset{\wedge }{X}}_{i}$ denotes the real trajectory value, and *X_i_* denotes the predicted trajectory value.

*FDE* refers to the mean square difference between the coordinates of the target box centroid, its width and height, and the position coordinates of the real trajectory, alongside the width and height of the target box at the last step of the predicted trajectory. It can be calculated as follows.

(13) $$FDE=\frac{1}{N}\sum_{i=1}^{N}\left\|\overset{\wedge }{X_{i}}-X_{i}\right\|\times 100$$

where *FDE* is the final displacement error.

Table 5 shows the evaluation results of this proposed trajectory prediction algorithm compared to the benchmark network on the D^2^-City dataset.

On the basis of the traditional S-GAN framework, the proposed trajectory prediction algorithm has added both a lane information fusion module and a trajectory adjustment module based on foreseeable information. Consequently, the prediction results of the proposed trajectory prediction algorithm exhibit significant improvement compared to the traditional S-GAN. Specifically, the ADE has decreased from 1.64 to 1.57, marking a reduction of 4.27% relative to the traditional S-GAN. Additionally, the FDE has decreased from 2.92 to 2.70, indicating a reduction in the final position offset error of 7.53% relative to the traditional S-GAN, which is a large improvement in the long-distance prediction performance. It indicates that the longer the time, the more the target’s trajectory relies less on its historical trajectory, and the lane information can improve this situation well. This ability of long-range prediction can alleviate the pressure on hardware to some extent.

Table 6 illustrates the impact of different prediction lengths on the prediction results under the same observation length. In this table, the observation length is fixed at five frames, while the prediction length varies between 5, 10, and 15 frames, respectively. It is evident that the error is relatively small when the prediction length is five frames, and trajectory prediction achieves a high accuracy rate by relying on the historical trajectory. However, as the prediction length increases to 10 frames, the *ADE* rises from 1.13 to 1.57, marking a 38.94% increase, and the *FDE* increases from 1.81 to 2.70, indicating a 49.17% increase compared to the 5-frame prediction length. Subsequently, when the prediction length extends to 15 frames, the *ADE* increases by 13.38%, and the *FDE* increases by 22.96% compared to the 10-frame prediction length. It indicates that the integration of lane information and forecasting data begin to have an effect, resulting in a lower growth rate of prediction errors.

**Figure 10:**
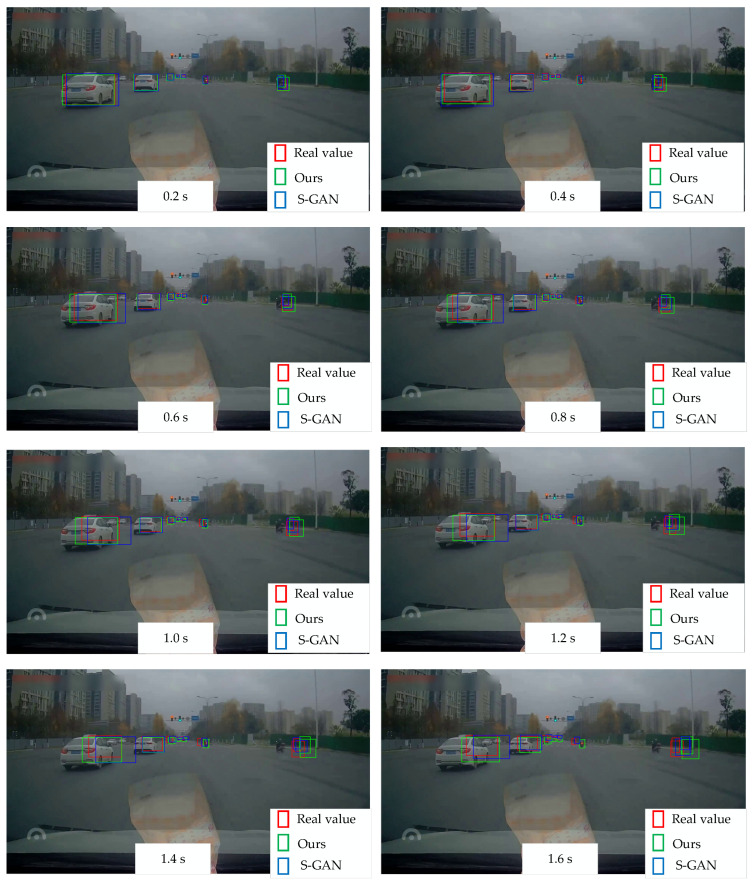
Comparison of trajectory prediction results. The prediction time is 0.2 s, 0.4 s, 0.6 s, 0.8 s, 1.0 s, 1.2 s, 1.4 s, and 1.6 s, respectively. The red box represents the real value, the green box represents the prediction result of the proposed trajectory prediction method, and the blue box represents the prediction result of the traditional S-GAN.

Figure 10 depicts a visual comparison of prediction results under urban road conditions between the proposed trajectory prediction algorithm and the traditional S-GAN. In the first two frames, the disparity between the two prediction methods and the actual values is not large. It can be seen that when the prediction step is short, trajectory prediction primarily relies on historical trajectory information. However, in the last six frames, the prediction results of the traditional S-GAN show an increasing deviation, notably with an obvious prediction error observed in the nearest left vehicle. This discrepancy arises primarily because the prediction is solely based on historical trajectory. Consequently, if the predicted vehicle is moving at high speed, it may lead to the prediction result shifting to the right, thereby influencing the host vehicle’s decision-making and normal driving behavior, potentially leading to traffic accidents. In contrast, the prediction results of the proposed trajectory prediction algorithm are more accurate, with minimal difference between the predicted and actual frames. While the detection of the real target is affected by the picture boundary, the prediction result accurately reflects the size of the entire vehicle. However, the prediction accuracy is lower for the motorcycle on the right side, possibly due to the motorcycle’s route being less influenced by lane information and exhibiting characteristics of sudden acceleration, which poses a challenge for the algorithm to handle.

## 6. Conclusions

For lane detection, a lane detection algorithm named CA-HDC is developed based on the traditional PINet to extract richer feature information. A channel attention mechanism is applied to the last layer of channels in each module, enriching the feature information to contain a wider range of detailed and semantic information. The average F1-measure has been increased by 4.1%, while the F1-measure for the shadow scenario has improved by 15.4% compared to the original PINet. Overall, the lane detection algorithm can effectively improve the detection accuracy.

On the base of the traditional S-GAN framework, the proposed multi-object trajectory prediction algorithm has added a lane information fusion module and a trajectory adjustment module based on foreseeable information. The proposed method enables the network to consider the high-level semantic information surrounding the target more fully, thereby improving prediction capability and reducing prediction algorithm error rates. Prediction performance is evaluated by using *ADE* and *FDE*. Compared to the traditional S-GAN, the *ADE* has been reduced by 4.27%, and the *FDE* has been reduced by 7.53%. The proposed trajectory prediction algorithm significantly enhances the prediction performance.

Although this paper has achieved promising results in lane detection and multi-object trajectory prediction, there is still room for improvement. For lane detection, further research is needed on how to recognize multi-lanes that may be obstructed, completely disappear, or suddenly increase within a certain period of time. Regarding trajectory prediction, this paper does not consider the intention of the host vehicle when adding information from other targets. It can be considered by adding such clues when constructing the dataset, which might positively impact the prediction results. In addition, the multi-objective trajectory prediction algorithm mainly uses LSTM for sequence-to-sequence prediction without testing the effect of other advanced neural networks, such as GRU, graph neural network, etc. In the future, the effect of these neural networks can be verified for comparison and to further optimize the model.

## Figures and Tables

**Table 1:** Detail of the lane detection network.

Name	Module	Type	Input Channel	Output Channel	Size/Step
Resizing layer	/	General convolutional	3	64	7/2
	CA-HDC	Same bottleneck layer/HDC	64/32	64/32	/
		Channel attention	/	/	/
	/	Maximum pooling layer	64	64	2/2
	CA-HDC	Same bottleneck layer/HDC	64/32	64/32	/
		Channel attention	/	/	/
	/	Maximum pooling layer	64	64	2/2
	CA-HDC	Same bottleneck layer/HDC	64/64	64/64	/
		Channel attention	/	/	/
Hourglass network	/	Downsampling bottleneck layer	128	128	/
	/	Downsampling bottleneck layer	128	128	/
	/	Downsampling bottleneck layer	128	128	/
	/	Downsampling bottleneck layer	128	128	/
	/	Same bottleneck layer	128	128	/
	/	Same bottleneck layer	128	128	/
	/	Upsampling bottleneck layer	128	128	/
	/	Upsampling bottleneck layer	128	128	/
	/	Upsampling bottleneck layer	128	128	/
	/	Upsampling bottleneck layer	128	128	/
Confidence branch	/	Same bottleneck layer	128	1	/
Offset branch	/	Same bottleneck layer	128	2	/
Feature branch	/	Same bottleneck layer	128	4	/

**Table 2:** Detail of lane information extraction backbone network.

Type	Number of Convolutional Kernel	Size of Convolutional Kernel	Step	Size of Feature Map
General convolutional	8	3 × 3	2	256 × 128
BN + Relu	/	/	/	/
General convolutional	8	5 × 5	4	64 × 32
BN + Relu	/	/	/	/
General convolutional	8	3 × 3	2	32 × 16
BN + Relu	/	/	/	/
Maximum pooling layer	8	2	2	16 × 8
Fully connected layer	/	/	/	/

**Table 3:** Characteristics of the CULane dataset.

Scenario	Number	Proportion
Normal	36,906	27.7%
Crowded	31,177	23.4%
Dazzle light	1865	1.4%
Shadow	3597	2.7%
No line	15,589	11.7%
Arrow	3464	2.6%
Curve	1599	1.2%
Night	27,047	20.3%
Crossroad	11,991	9.0%
Total	133,235	100%

**Table 4:** F_1_-measure result on the CULane dataset.

Scenario	PINet [10]	Ours	Enhancement
Norman	87.5%	88.2%	0.8%
Crowded	68.2%	70.1%	2.8%
Dazzle light	62.4%	62.8%	0.6%
Shadow	61.0%	70.4%	15.4%
No line	45.5%	47.3%	4.0%
Arrow	79.1%	82.2%	4.8%
Curve	63.1%	63.7%	1.0%
Night	64.4%	67.7%	5.1%
Average	66.4%	69.1%	4.1%

**Table 5:** Evaluation result on the D^2^-City dataset.

Algorithm	Observation Length (Frame)	Prediction Length (Frame)	*ADE*	*FDE*
Ours	5	10	1.57	2.70
Traditional S-GAN [14]	5	10	1.64	2.92
Enhancement	/	/	4.27%	7.53%

**Table 6:** Influence of different prediction lengths.

Observation Length (Frame)	Prediction Length (Frame)	*ADE*	*FDE*
5	5	1.13	1.81
	10	1.57	2.70
	15	1.78	3.32

## Data Availability

The data that support the findings of this study are available from the corresponding authors upon request.
